# Mortality from Ischemic Heart Disease and Diabetes Mellitus (Type 2) in Four U.S. Wheat-Producing States: A Hypothesis-Generating Study

**DOI:** 10.1289/ehp.8352

**Published:** 2005-10-06

**Authors:** Dina M. Schreinemachers

**Affiliations:** National Health and Environmental Effects Research Laboratory, Office of Research and Development, U.S. Environmental Protection Agency, Research Triangle Park, North Carolina, USA

**Keywords:** chlorophenoxy herbicides, clofibrate, coronary atherosclerosis, C-reactive protein, diabetes, ischemic heart disease, myocardial infarction

## Abstract

In this ecologic study I examined ischemic heart disease (IHD) and diabetes mortality in rural agricultural counties of Minnesota, Montana, North Dakota, and South Dakota, in association with environmental exposure to chlorophenoxy herbicides, using wheat acreage as a surrogate exposure. I collected data on agricultural land use and 1979–1998 mortality from the U.S. Department of Agriculture and the Centers for Disease Control and Prevention websites, respectively. Counties were grouped based on percentage of land area dedicated to wheat farming. Poisson relative risks (RR) and 95% confidence intervals (CIs), comparing high- and medium- with low-wheat counties, were obtained for IHD, the subcategories acute myocardial infarction (AMI) and coronary atherosclerosis (CAS), and diabetes, adjusting for sex, age, mortality cohort, and poverty index. Mortality from IHD was modestly increased (RR = 1.08; 95% CI, 1.04–1.12). Analyses of its two major forms were more revealing. Compared with low-wheat counties, mortality in high-wheat counties from AMI increased (RR = 1.20; 95% CI, 1.14–1.26), and mortality from CAS decreased (RR = 0.89; 95% CI, 0.83–0.96). Mortality from AMI was more pronounced for those < 65 years of age (RR = 1.31; 95% CI 1.22–1.39). Mortality from type 2 diabetes increased (RR = 1.16; 95% CI, 1.08–1.24). These results suggest that the underlying cause of mortality from AMI and type 2 diabetes increased and the underlying cause of mortality from CAS decreased in counties where a large proportion of the land area is dedicated to spring and durum wheat farming. Firm conclusions on causal inference cannot be reached until more definitive studies have been conducted.

A series of International Agency for Research on Cancer multinational studies of workers involved in the production of chlorophenoxy herbicides and chlorophenols indicated excess mortality from cancer, ischemic heart disease (IHD), and possibly diabetes in association with 2,3,7,8-tetrachlorodibenzo-*p*-dioxin (TCDD) exposure (Flesch-Janys et al. 1995; Hooiveld et al. 1998; Kogevinas et al. 1997; Vena et al. 1998). Chemical production workers exposed to TCDD are simultaneously exposed to much higher levels of the commercial chemicals being produced, such as chlorophenoxy herbicides and chlorophenols (Remillard and Bunce 2002). Results from the cancer mortality study among chlorophenoxy herbicide production workers, some of whom were exposed to TCDD or higher chlorinated dioxins (Kogevinas et al. 1997), were similar to those reported in an ecologic mortality study of cancer among residents in rural, agricultural counties of Minnesota, Montana, North Dakota, and South Dakota potentially exposed to chlorophenoxy herbicides and/or contaminants (Schreinemachers 2000). This similarity of results led to the question of whether increased mortality from IHD and diabetes observed among the chlorophenoxy herbicide production workers (Vena et al. 1998) might also be observed among residents of agricultural counties of Minnesota, Montana, North Dakota, and South Dakota, where the major field crops spring and durum wheat have been treated predominantly and long term with chlorophenoxy herbicides (Lin et al. 1995).

The chlorophenoxy herbicides 2,4-di-chlorophenoxyacetic acid (2,4-D) and 4-chloro-2-methylphenoxyacetic acid (MCPA) have been widely applied in the United States since World War II and are used for broadleaf weed control in wheat farming and maintenance of home lawns and parks, rights-of-way, and road sides (Short and Colborn 1999). 2,4-D used for home lawn maintenance is likely to be found in residential carpet dust up to 1 year after application (Nishioka et al. 1996). Chlorophenoxy herbicides are present in agricultural and urban streams and in the atmosphere [U.S. Geological Survey (USGS) 1995, 2003]. 2,4-D and MCPA are transported over short and long distances attached to air particles, with concentrations highest in regions where they are applied at the time of application (Waite et al. 2002, 2005). Contaminants present in technical-grade 2,4-D and MCPA include polychlorinated dibenzo-dioxins and dibenzofurans and 2,4-dichloro-phenol [U.S. Department of Agriculture (USDA) 1998]. More recently, aryl hydrocarbon receptor–based assays for dioxin-like activity of chlorinated herbicides applied in the Minnesota Red River Valley showed that most of these commercial-grade mixtures had measurable dioxin-like activity (Huwe et al. 2003). Confirmatory analytic chemical studies showed that some of the 2,4-D ester formulations were contaminated with dioxins/furans, including trace amounts of TCDD.

Residents of agricultural counties of Minnesota, Montana, North Dakota, and South Dakota may be environmentally and/or occupationally exposed to 2,4-D and MCPA. Wheat acreage per county or percentage of the county’s land area dedicated to wheat farming was used in two previous ecologic studies as a surrogate measure of exposure to chlorophenoxy herbicides and/or contaminants, because information on herbicide use by county was not available (Schreinemachers 2000, 2003). Use of this surrogate exposure measure was a reasonable choice because chlorophenoxy herbicides are the predominant herbicides applied to wheat and because other major field crops in these four states, corn and soybeans, are mostly treated with other herbicides, based on information on herbicide use by crop, state, and year, available since 1991 (USDA 1991). Before 1991, information on herbicide use was available only for groups of states.

In the present ecologic study I investigated the possible links between environmental exposures to chlorophenoxy herbicides and mortality during 1979–1988 and 1989–1998 from IHD and type 2 diabetes. Counties with different levels of wheat farming provided a gradient of exposures, thereby overcoming the lack of a null referent. This hazard identification study can be one of the initial steps in defining an association between environmental exposures to chlorophenoxy herbicides and mortality from IHD and diabetes.

## Materials and Methods

### Mortality.

I obtained data on underlying cause of death based on the *International Classification of Diseases, 9th Revision* (ICD-9) (U.S. Department of Health and Human Services 1989) by state, county, sex, and age group for 1979–1988 and 1989–1998 for Minnesota, Montana, North Dakota, and South Dakota [Centers for Disease Control and Prevention (CDC) 2004a]. Deaths were distributed by 5-year age groups for mortality < 25 years of age and by 10-year age groups for mortality ≥ 25 years of age. Mortality data for the following diseases were extracted: IHD (ICD-9 410–414.9), acute myocardial infarction (AMI; ICD-9 410), coronary atherosclerosis (CAS; ICD-9 414.0), and diabetes mellitus (all types, ICD-9 250.0–250.9). Population data were obtained from the Population Census (CDC 2003). Only white subjects were included because the percentage of nonwhites in these states (< 10%) was too small to support reliable analyses. This study analyzed publicly available data sets and was exempt from institutional review board approval.

### Agricultural data.

From the combined 262 counties of the four states, agricultural counties with a mostly rural population were selected based on the criteria of ≥ 20% of county’s land area dedicated to cropland and ≥ 50% of population defined as rural (U.S. Census Bureau 1980a; USDA 1982). Rural populations living in agricultural counties are more likely to be exposed to agricultural chemicals than are urban populations. Farming communities tend to be more residentially stable than urban communities (Blair and Zahm 1995). Although farmers and their families may have been exposed to higher levels of pesticides than the general rural population, it was not possible to distinguish between farmers and nonfarmers. Averages of total wheat acreage per county were determined for 1970–1979, 1980–1989, and 1990–1999, based on annual estimates of total wheat acreages per county (USDA 2004). Wheat acreage for 1964 was obtained from the 1964 USDA Agricultural Census (USDA 1964).

To further define wheat acreage in rural, agricultural counties of Minnesota, Montana, North Dakota, and South Dakota as a surrogate for chlorophenoxy herbicide use, I determined acreage for the different classes of wheat in the selected counties. The percentage of herbicide-treated acreage for spring and durum wheat is larger than for winter wheat, which is a better competitor with weeds (Lin et al. 1995). For counties with a large winter wheat acreage, I applied a correction factor to the winter wheat acreage before combining it with spring and durum wheat acreage to obtain a value for total wheat acreage. This correction factor was based on 1991–1998 herbicide treatment for the different classes of wheat (USDA 1991). This information was not available for years before 1991.

### Statistical methods.

I used Spearman correlations to determine the associations among 1964 and average 1970–1979, 1980–1989, and 1990–1999 levels of wheat acreage. Depending on the number of deaths available for the specific underlying cause under study, counties were grouped based on the median or tertiles of the percentage of land area dedicated to wheat. For diabetes mortality, I compared high-wheat counties with low-wheat counties. For mortality from IHD, including AMI and CAS, I compared high- and medium-wheat counties with low-wheat counties. For the univariate analyses by mortality cohort, sex, and age groups, I calculated standardized rate ratios (SRRs) and 95% confidence interval (CI) using direct age standardization based on the 1970 U.S. population, according to established methods (Greenland and Rothman 1998). For the multivariate analyses I used Poisson regressions, adjusting for mortality cohort (1989–1998 vs. 1979–1988), sex (male vs. female), age (≥ 65 vs. < 65 years of age), and county’s poverty level (based on median percentage of families with income below poverty level in 1979, ≥ 13.15 vs. < 13.15 [U.S. Census Bureau 1980b]). Analyses for IHD mortality excluded subjects < 25 years of age. Analyses for diabetes mortality included subjects ≥ 45 years of age so that the obtained results refer mostly to type 2 diabetes (Calle et al. 1998). The high number of deaths from AMI made it possible to include additional analyses. Separate Poisson models were run for subjects < 65 years and ≥ 65 years of age. I estimated the wheat effect on AMI mortality for individual states by comparing high- with low-wheat counties based on the median wheat percentage of each state. Statistical analyses and creation of figures were performed using SAS software (SAS Institute Inc. 2001).

## Results

### Wheat and chlorophenoxy herbicide use.

Although the average percentage of a county’s land area dedicated to wheat increased from 6.9% in 1964 to 12.1% during 1990–1999, Spearman correlations for the average 1970–1979 wheat acreage with the 1964, average 1980–1989, and average 1990–1999 wheat acreages were 0.98 (*p* < 0.0001), 0.95 (*p* < 0.0001), and 0.94 (*p* < 0.0001), respectively. This implied that a county with a high percentage of its land area dedicated to wheat during 1970–1979 was most likely also a high-wheat county during 1964, 1980–1989, and 1990–1999. The average total wheat acreage for 1970–1979 expressed as a percentage of a county’s land area was used as a surrogate measure of exposure to chlorophenoxy herbicides. Wheat acreage during the other time periods could probably also have been used given the high correlations. A USDA report showed that in 1976, > 90% of the herbicides applied to wheat in the United States consisted of chlorophenoxy herbicides (Eichers et al. 1978). The percentage of chlorophenoxy herbicides applied overall to wheat decreased to 67% in 1992 because of increased use of other herbicides (Lin et al. 1995).

I determined acreage for the different classes of wheat in the selected counties of the four states for 1970–1979. The estimated acreage of winter wheat during 1970–1979 was < 2% in the selected counties of Minnesota and North Dakota, so total wheat acreage was practically synonymous with the combined durum and other spring wheat acreage. However, the average acreage of winter wheat in the selected Montana and South Dakota counties contributed 36% and 20%, respectively, to the total wheat acreage. Given these data, it seemed appropriate to apply a correction factor to winter wheat acreage in Montana and South Dakota to account for the less intensive herbicide treatment. The amount of herbicides used on different classes of wheat has been made available for individual states since 1991. The 1991–1998 average percentage of durum and other spring wheat acreage treated with any herbicides in Minnesota, Montana, North Dakota, and South Dakota was 91%, whereas the average percentages of winter wheat in Montana and South Dakota treated with any herbicide were 86% and 67%, respectively. Assuming that the percentage of herbicide treated winter wheat acreage in Montana and South Dakota during 1991–1998 was similar to the percentage of herbicide-treated acreage during 1970–1979, a correction was applied to the 1970–1979 winter wheat acreage in counties of Montana and South Dakota, by multiplying the winter wheat acreage by 0.95 (86/91) and 0.74 (67/91), respectively, before combining winter wheat acreage with durum and other spring wheat acreage by county to obtain a value for adjusted total wheat acreage.

### County characteristics.

Characteristics of the 152 selected counties distributed over three groups based on tertiles of the counties’ wheat percentage are presented in Table 1. Most counties in the low-wheat group were located in Minnesota, whereas counties in the high-wheat group were mostly located in North Dakota. Medium- and high-wheat counties grew less corn and soybeans than did low-wheat counties and had a larger rural and farm population size. With increasing wheat percentage, the total population size decreased, whereas the percentage of subjects ≥ 65 years of age increased.

### Cardiovascular disease and diabetes mortality.

Table 2 presents crude and age-adjusted mortality rates for all ages by sex and mortality cohort. Age-adjusted U.S. rates are also presented for comparison (CDC 2004a). U.S. rates for mortality from IHD and diabetes were slightly higher than rates in the combined selected counties. Comparison of the two cohorts showed that 1989–1998 mortality from diabetes (all types) increased > 20%, whereas mortality from IHD decreased > 30%, which is consistent with the overall decline of mortality from IHD (including AMI and CAS) due to improved dietary patterns and treatment, decreased smoking, and increased physical activity (CDC 1992, 1997). Analysis of the two main subcategories of IHD revealed that age-adjusted rates for AMI among men were approximately 10% higher, and rates of CAS for both men and women were > 20% lower, in selected counties compared with the U.S. population.

The results from Poisson models adjusting simultaneously for mortality cohort, sex, age, and poverty are presented in Table 4. In high-wheat counties, mortality from IHD, AMI, and diabetes (mostly type 2 because of excluding deaths of subjects < 45 years of age) was significantly increased by 8, 20, and 16%, respectively, whereas mortality from CAS was significantly decreased by 11% (models 1–4). The mortality increase from AMI in high-wheat counties was more pronounced among subjects < 65 years (31%) than for those ≥ 65 years of age (16%) (models 5–6). Given that chlorophenoxy herbicides use started around 1950, those that died < 65 years of age may have been exposed prenatally and/or as a child. Mortality from AMI for individual states (models 7–10) was significantly increased in Minnesota (12%), North Dakota (16%), and South Dakota (7%). The effect for Montana (6%) was not significant, which was likely due to the low number of deaths from AMI and the few and narrow range of counties. To test this possibility, I ran a separate model for Minnesota, restricted to counties in the same wheat percentage range as Montana, which resulted in a nonsignificant wheat effect of 1.05 (0.91–1.15), similar to the results obtained for Montana.

I repeated the Poisson models after applying a correction factor based on the winter wheat acreage in Montana and South Dakota. The adjusted results were very similar to the nonadjusted results (data not shown).

## Discussion

The observed increases in mortality from IHD and diabetes in the general rural population of agricultural counties in Minnesota, Montana, North Dakota, and South Dakota are consistent with results from studies on effects from exposure to dioxin and dioxin-like compounds. Chlorophenoxy herbicides and chlorophenol production workers exposed to dioxin, based on estimates from job records and company exposure questionnaires, showed an increase in mortality from IHD and possibly diabetes (Vena et al. 1998). Likewise, studies of Operation Ranch Hand veterans showed an increase of mortality from circulatory system diseases (Michalek et al. 1998) and an increase in diabetes prevalence (Henriksen et al. 1997). This association between diabetes and serum dioxin levels in the Vietnam veterans has been defined as “limited and suggestive” (Brown 2000). A dose response of IHD mortality in association with exposure to polychlorinated dibenzo-*p*-dioxins and dibenzofurans was observed in a German herbicide-producing plant (Flesch-Janys et al. 1995). Widespread exposure to dioxin from the Seveso, Italy, accident was associated with increased mortality from chronic IHD, cancer, and diabetes (Bertazzi et al. 2001). Increased hospitalization rates for IHD were observed among New York State residents living near sites contaminated with persistent organic pollutants (Sergeev and Carpenter 2005). Increased mortality from diabetes and several cancers has been observed for pulp and paper mill workers (Axelson et al. 1998; Henneberger et al. 1989; Schwartz 1988; Wingren et al. 1991). Environmental factors are thought to play a role (Carpenter et al. 2002; Longnecker and Daniels 2001; Remillard and Bunce 2002).

The increase in mortality from type 2 diabetes with increasing wheat acreage should be considered with caution. Estimated rates of diabetes mortality may be unreliable because of severe underreporting of the disease (CDC 2004b), in addition to usually not being listed as underlying cause of death (Geiss et al. 1995). To support the diabetes results, mortality in high- and low-wheat counties were compared for two additional diseases known to be associated with diabetes, namely, renal disease (ICD-9 580–589) and cerebrovascular disease (ICD-9 430–438) (CDC 2004b; Harris 1995). Mortality from renal disease increased by 21% in high-wheat counties [risk ratio (RR) = 1.21; 95% CI, 1.12–1.31], whereas mortality from cerebrovascular disease did not show an effect (RR = 0.98; 95% CI = 0.94–1.03). These two disease groups were not further investigated.

The seemingly contradictory outcomes of increasing mortality from AMI and decreasing mortality from CAS in association with wheat acreage agree with results of a study on cardiovascular mortality during 1987–1997 in Minnesota (Morrison et al. 2000). This study showed that in the northwest region of Minnesota, where wheat is one of the major field crops, mortality from AMI was higher and mortality from CAS was lower compared with the other Minnesota regions, both in younger and older men and women. Assuming that CAS is a major risk factor for AMI, one could argue that among subjects susceptible to IHD, those more highly exposed died from AMI as underlying cause, resulting in fewer deaths being available for CAS, a case of competing mortality. Other interpretations are also worth considering. AMI often occurs in the absence of hyperlipidemia. Recent studies have shown that high levels of C-reactive protein (C-RP), an indicator of systemic inflammation, are associated with increased risk of AMI (as well as metabolic syndrome and type 2 diabetes), independent of the level of CAS (Pradhan et al. 2001; Ridker et al. 2004). Although increased levels of C-RP and atherosclerosis may indicate distinct risk groups (Theuma and Fonseca 2003), elevated C-RP levels are thought to be the stronger predictor of future cardiovascular events and may be associated with plaque fragility and rupture (Aronow 2003; Hansson 2005; Ridker et al. 2002, 2004). If the increased mortality observed for AMI in the present study is indeed associated with increased C-RP levels, the observed decrease in mortality from CAS may now have an alternative interpretation. Considering that 2,4-D and MCPA have similar chemical structures as the hypolipidemic drug clofibrate (2-[4-chlorophenoxy]-2-methylpropionic acid ethyl ester) (Axelson et al. 1980) and that 2,4-D and MCPA, as well as clofibrate, have lipid-lowering effects in rats (Vainio et al. 1983), one might ask if the observed decrease in mortality from CAS could be caused by a lipid-lowering effect from environmental exposures to chlorophenoxy herbicides. Further studies will have to investigate this. Finally, differences among counties in determination of underlying cause of death as well as chance may have contributed to these findings.

Conducting investigations of associations between environmental exposures and adverse human health effects is difficult for several reasons. Exposures are most likely widespread and low dose. A null comparison group may be unavailable (damage to the environment is global). Long lag periods between time of exposure and time of diagnosis of the chronic disease under study may present problems (McMichael 2002). Use of a multilevel approach may be warranted (Pekkanen and Pearce 2001; Susser 1998). Evidence from studies at the molecular, individual, population, and/or ecosystem level needs to be combined to completely define the link between environmental exposure and health effects. This requires multidisciplinary studies and interdisciplinary collaborations. Population (or ecologic) studies are fundamental as a first step in identifying a potential hazard and defining the key public health problem, because effects from exposures common throughout a study population can be uncovered only by comparison of populations (Pekkanen and Pearce 2001). Results from a single ecologic study can be easily misinterpreted and cannot establish causal inference (Morgenstern 1995). Individual risk factors cannot be accounted for in an ecologic study, because the focus is on the environment in which people live rather than on their personal lifestyles. Therefore, an ecologic study needs to be followed by an individual risk factor study based on hypotheses generated by the ecologic results, with adjustment for individual risk factors and confounders. An example in this study would be the potential confounding effect from smoking. Controlling for smoking usually has only a modest effect if risk estimates are high (Axelson and Steenland 1988). Although in the present study risk estimates for AMI are relatively low, there is no reason to assume that smoking rates per county increase with intensity of wheat farming. The fact that mortality from AMI increases with wheat percentage for both sexes, both younger and older subjects, and both 1979–1988 and 1989–1998 mortality cohorts suggests that the effects are associated with wheat agriculture.

The present ecologic study as well as several previous studies used existing databases (Garry et al. 1996; Schreinemachers 2000, 2003; Schreinemachers et al. 1999). Comparison of regions at different levels of wheat farming provided the opportunity to observe adverse health effects in association with environmental exposures to chlorophenoxy herbicides and/or contaminants. Individual risk factor analysis could not have uncovered these associations easily. Results from the present study have generated hypotheses concerning the increase of AMI and the decrease of CAS in association with environmental, repeated (annually), low-dose exposures to chlorophenoxy herbicides and/or contaminants. Future, more definitive studies should include biomarkers such as serum levels of dibenzodioxins and dibenzofurans, levels of glycosylated hemoglobin, lipid levels, white blood cell counts and determination of C-RP. Subject-based studies should adjust for known individual risk factors for the diseases under study, such as obesity, smoking, socioeconomic factors, and access to medical care. Molecular studies could investigate whether chlorophenoxy herbicides are synthetic ligands for peroxisome proliferator activated receptors (PPARs). Activation of PPARs is the mechanism by which hypolipidemic fibrates induce hypolipidemia in humans (Clay et al. 2000; Vamecq and Latruffe 1999).

In summary, this ecologic study is an example of how population studies can make valuable contributions to public health by identifying potential environmental hazards.

## Addendum

The population estimates used for the analyses were obtained from CDC WONDER and consist of Census intercensal estimates for 1971–1979 and 1981–1989, Census postcensal estimates for 1991–1999, and modified age–race–sex Census counts for the census years (1970, 1980, 1990, and 2000). These were the best population estimates available at the time of data collection (January–July 2003). Recent Census Bureau intercensal estimates for 1991–1999 showed that the postcensal estimates for 1991–1999 were too low. As a result, in the current study, the 1989–1998 population was underestimated by 0.6%. However, reanalysis based on the more accurate population data showed that this had negligible effects on the results (data not presented).

## Figures and Tables

**Figure 1:**
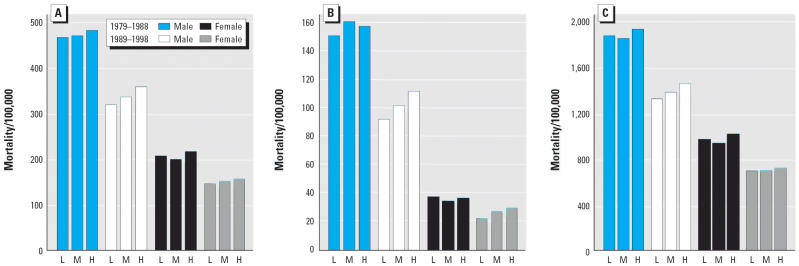
IHD (ICD-9 410–414.9): Age-standardized mortality rates/100,000 (underlying cause) for grouped counties based on tertiles of percentage of a county’s land area dedicated to wheat. Comparison of high-wheat (H) and medium-wheat counties (M) with low-wheat counties (L), by age group, sex, and mortality cohort. (*A*) Age 25– ≥ 85 years; (*B*) Age 25–64 years; (*C*) Age 65– ≥ 85 years.

**Figure 2:**
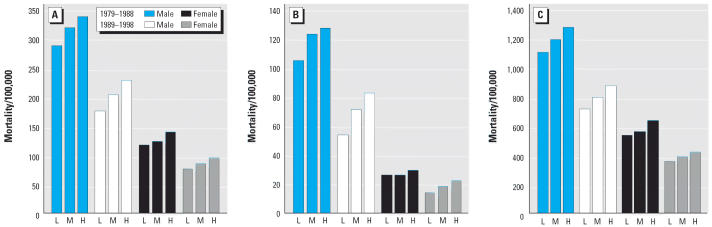
AMI (ICD-9 410): Age-standardized mortality rates/100,000 (underlying cause) for grouped counties based on tertiles of percentage of a county’s land area dedicated to wheat. Comparison of high-wheat (H) and medium-wheat counties (M) with low-wheat counties (L), by age group, sex, and mortality cohort. (*A*) Age 25– ≥ 85 years; (*B*) Age 25–64 years; (*C*) Age 65– ≥ 85 years.

**Figure 3:**
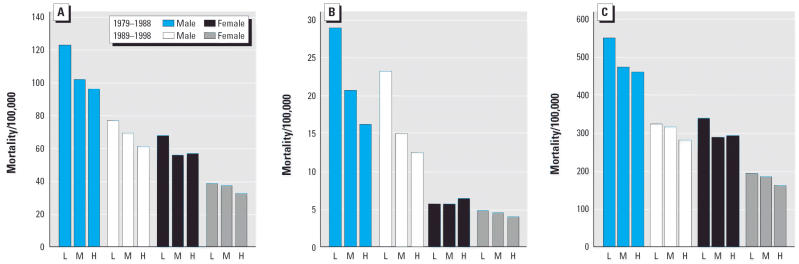
CAS (ICD-9 414.0): Age-standardized mortality rates/100,000 (underlying cause) for grouped counties based on tertiles of percentage of a county’s land area dedicated to wheat. Comparison of high-wheat (H) and medium-wheat counties (M) with low-wheat counties (L), by age group, sex, and mortality cohort. (*A*) Age 25– ≥ 85 years; (*B*) Age 25–64 years; (*C*) Age 65– ≥ 85 years.

**Figure 4:**
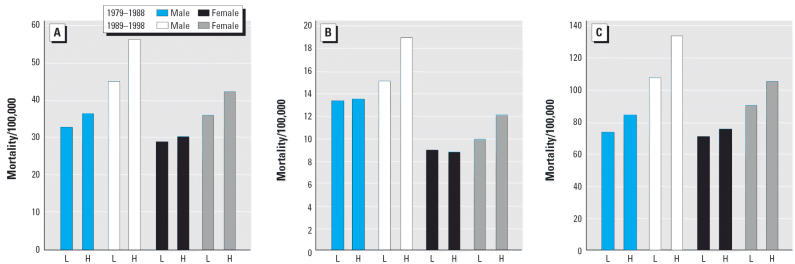
Diabetes mellitus (ICD-9 250.0–250.9): Age-standardized mortality rates/100,000 (underlying cause) for grouped counties based on median of percentage of a county’s land area dedicated to wheat. Comparison of high-wheat counties (H) with low-wheat counties (L), by age group, sex, and mortality cohort. (*A*) Age 45– ≥ 85 years; (*B*) Age 45–64 years; (*C*) Age 65– ≥ 85 years.

**Table 1:** Characteristics [median (range)] of three groups of counties based on tertiles of 1970–1979 percentage wheat acreage/county.

	Average 1970–1979 percent wheat acreage per county		
Characteristics of county groups	Low 0.95 (0.09–4.00)	Medium 8.43 (4.04–14.32)	High 22.91 (14.52–35.47)
No. of selected counties			
Minnesota	32	14	10
Montana	1	8	6
North Dakota	0	10	30
South Dakota	18	19	4
Total	51	51	50
County land area, acres × 10^6^	0.37 (0.22–1.92)	0.71 (0.33–3.16)	0.71 (0.28–2.55)
County cropland, acres × 10^6^	0.24 (0.07–0.51)	0.34 (0.15–0.71)	0.50 (0.18–1.24)
1970–1979 percent dedicated land			
Corn	21.74 (0.00–39.89)	0.93 (0.00–31.98)	0.06 (0.00–21.59)
Soybeans	4.16 (0.00–38.98)	0.00 (0.00–28.87)	0.01 (0.00–10.25)
Farm size, 1982 (acres)	303 (147–4,726)	1,276 (233–5,154)	984 (453–3,661)
Percent rural population, 1982	79.4 (53.1–100)	100 (52.2–100)	100 (50.7–100)
Percent farm population, 1982	25.1 (6.2–48.6)	30.5 (11.9–64.8)	30.2 (13.9–51.1)
Percent families below poverty level, 1979	12.0 (4.7–38.0)	14.9 (7.5–34.3)	12.7 (6.6–25.7)
Average annual population at risk (percent ≥ 65 years)			
1979–1988			
Male	447,124 (12.5)	220,924 (15.1)	172,776 (16.2)
Female	452,703 (16.5)	220,884 (18.8)	171,621 (20.0)
1989–1998			
Male	479,003 (12.5)	209,192 (16.3)	149,245 (18.1)
Female	482,825 (16.6)	210,840 (20.6)	150,107 (22.9)

**Table 2:** Mortality rates of IHD, AMI, CAS, and diabetes mellitus for all ages in rural, agricultural counties of Minnesota, Montana, North Dakota, and South Dakots, compared with U.S. rates.

		Selected counties (*n* = 152)			
			Rate/100,000		
Underlying cause (ICD-9)	Year of death	No.	Crude	Age adjusted	U.S. (white) rate/100,000 age adjusted
IHD (410–414.9)					
Male	1979–1988	28,599	340.1	250.7	272.2
	1989–1998	21,763	259.9	176.4	189.1
Female	1979–1988	19,651	232.5	110.6	138.2
	1989–1998	16,821	199.4	79.8	100.9
AMI (410)					
Male	1979–1988	18,338	218.1	165.0	150.6
	1989–1998	12,493	149.2	104.0	92.5
Female	1979–1988	10,996	130.1	66.9	68.8
	1989–1998	8,663	102.7	44.3	47.3
CAS (414.0)					
Male	1979–1988	7,318	87.0	59.9	88.1
	1989–1998	5,032	60.1	38.9	50.1
Female	1979–1988	6,831	80.8	33.5	54.8
	1989–1998	4,821	57.1	20.2	31.6
Diabetes mellitus (250)					
Male	1979–1988	1,235	14.7	10.9	12.7
	1989–1998	1,888	22.5	15.4	17.0
Female	1979–1988	1,453	17.2	9.3	11.6
	1989–1998	2,148	25.5	12.0	14.0
Person-years at risk					
Male	1979–1988	8,408,234			
	1989–1998	8,374,404			
Female	1979–1988	8,452,082			
	1989–1998	8,437,721			

**Table 4:** Poisson regression comparing high-wheat (HW) and medium-wheat (MW) counties with low-wheat counties (LW), with adjustment for mortality cohort, sex, age, and poverty index.

Model no., disease (ICD-9)	Age (years)	RR (95% CI)	No.	(Male/female)
Combined states, combined age groups				
1. IHD (410–414.9)	25– ≥ 85			
LW		1.00	42,444	(24,139/18,305)
MW		1.01 (0.98–1.04)	23,780	(14,112/9,668)
HW		1.08 (1.04–1.12)	20,592	(12,099/8,493)
2. AMI (410)	25– ≥ 85			
LW		1.00	23,359	(14,016/9,343)
MW		1.09 (1.04–1.14)	14,293	(8,925/5,368)
HW		1.20 (1.14–1.26)	12,828	(7,884/4,944)
3. CAS (414.0)	25– ≥ 85			
LW		1.00	12,732	(6,535/6,197)
MW		0.90 (0.85–0.96)	6,300	(3,271/3,029)
HW		0.89 (0.83–0.96)	4,967	(2,541/2,426)
4. Diabetes mellitus (250.0–250.9)	45– ≥ 85			
LW		1.00	4,251	(1,920/2,331)
HW		1.16 (1.08–1.24)	2,271	(1,083/1,188)
Combined states, separate age groups				
5. AMI (410)	25–64			
LW		1.00	3,705	(2,952/753)
MW		1.21 (1.14–1.29)	2,378	(1,924/454)
HW		1.31 (1.22–1.39)	2,018	(1,612/406)
6. AMI (410)	65– ≥ 85			
LW		1.00	19,654	(11,064/8,590)
MW		1.05 (1.00–1.11)	11,915	(7,001/4,914)
HW		1.16 (1.10–1.22)	10,810	(6,272/4,538)
Separate states, combined age groups				
AMI (410)	25– ≥ 85			
7. Minnesota, LW		1.00	16,337	(9,813/6,524)
Minnesota, HW		1.12 (1.06–1.19)	14,586	(8,978/5,608)
8. Montana, LW		1.00	939	(616/323)
Montana, HW		1.06 (0.91–1.23)	1,013	(616/397)
9. North Dakota, LW		1.00	3,282	(2,055/1,227)
North Dakota, HW		1.16 (1.08–1.24)	4,947	(3,162/1,785)
10. South Dakota, LW		1.00	4,757	(2,797/1,960)
South Dakota, HW		1.07 (1.00–1.15)	4,619	(2,788/1,831)

RR values are adjusted for sex (male vs. female), age (≥ 65 vs. < 65), mortality cohort (1989–1998 vs. 1979–1988), and poverty index (1980 median, ≥ 13.15 vs. < 13.15). The exposure variable, percentage of a county’s land area dedicated to wheat farming, is based on tertiles with cut-points 4.04 and 14.50 (models 1–3, 5, 6) or the median with cut-point 8.43 (model 4). Median wheat percentage values for the individual states are as follows: Minnesota, 3.19 (model 7); Montana, 9.87 (model 8); North Dakota, 19.93 (model 9); and South Dakota, 5.29 (model 10). Median for individual states’ poverty index are Minnesota, 11.00; Montana, 11.40; North Dakota, 13.20; and South Dakota, 19.30.

**Table 3:** Age-standardized mortality rates/100,000, ratios, and 95% CIs for IHD, AMI, CAS, and diabetes mellitus, in low-, medium-, and high-wheat counties.

					Medium wheat		High wheat	
Underlying cause of death (ICD-9)	Age (years)	Sex	Year of death	Low wheat rate	Rate	SRR (95% CI)	Rate	SRR (95% CI)
IHD (410–414.9)								
	25– ≥ 85	Male	1979–1988	458.00	463.55	1.01 (0.98–1.04)	475.52	1.04 (1.01–1.07)
			1989–1998	312.03	331.23	1.06 (1.03–1.10)	353.28	1.13 (1.09–1.17)
		Female	1979–1988	205.03	196.47	0.96 (0.92–0.99)	213.27	1.04 (1.00–1.08)
			1989–1998	143.93	148.76	1.03 (0.99–1.08)	154.43	1.07 (1.03–1.12)
	25–64	Male	1979–1988	148.30	158.30	1.07 (1.00–1.14)	155.32	1.05 (0.98–1.12)
			1989–1998	89.26	99.75	1.18 (1.03–1.21)	109.90	1.23 (1.13–1.34)
		Female	1979–1988	36.89	34.30	0.93 (0.82–1.06)	35.56	0.96 (0.84–1.10)
			1989–1998	22.27	26.91	1.21 (1.04–1.41)	29.56	1.33 (1.13–1.56)
	65– ≥ 85	Male	1979–1988	1844.07	1829.70	0.99 (0.96–1.02)	1908.59	1.03 (1.00–1.07)
			1989–1998	1309.05	1367.23	1.04 (1.01–1.08)	1442.56	1.10 (1.06–1.14)
		Female	1979–1988	957.57	922.24	0.96 (0.93–1.00)	1008.60	1.05 (1.01–1.09)
			1989–1998	688.44	694.10	1.01 (0.97–1.05)	713.32	1.04 (0.99–1.08)
AMI (410)								
	25– ≥ 85	Male	1979–1988	285.31	315.87	1.11 (1.07–1.15)	333.99	1.17 (1.13–1.21)
			1989–1998	173.68	202.03	1.16 (1.11–1.21)	225.77	1.30 (1.24–1.36)
		Female	1979–1988	117.25	123.06	1.05 (1.00–1.10)	138.32	1.18 (1.12–1.24)
			1989–1998	75.24	85.27	1.13 (1.07–1.20)	93.91	1.25 (1.18–1.32)
	25–64	Male	1979–1988	104.21	121.89	1.17 (1.09–1.26)	126.04	1.21 (1.12–1.31)
			1989–1998	53.11	71.20	1.34 (1.22–1.48)	82.16	1.55 (1.40–1.71)
		Female	1979–1988	25.54	26.06	1.02 (0.88–1.19)	28.58	1.12 (0.96–1.31)
			1989–1998	13.46	18.13	1.35 (1.12–1.63)	22.08	1.64 (1.35–1.99)
	65– ≥ 85	Male	1979–1988	1095.81	1184.03	1.08 (1.04–1.12)	1264.69	1.15 (1.11–1.20)
			1989–1998	713.32	787.56	1.10 (1.05–1.16)	868.54	1.22 (1.16–1.28)
		Female	1979–1988	527.69	557.16	1.06 (1.01–1.11)	629.51	1.19 (1.13–1.25)
			1989–1998	351.77	385.76	1.10 (1.04–1.16)	415.35	1.18 (1.11, 1.25)
CAS (414.0)								
	25– ≥ 85	Male	1979–1988	122.72	102.34	0.83 (0.79–0.88)	96.19	0.78 (0.74–0.83)
			1989–1998	77.44	69.49	0.90 (0.84–0.96)	61.05	0.79 (0.73–0.85)
		Female	1979–1988	67.54	56.16	0.83 (0.78–0.88)	56.89	0.84 (0.79–0.90)
			1989–1998	39.18	37.56	0.96 (0.89–1.03)	32.72	0.84 (0.77–0.91)
	25–64	Male	1979–1988	28.64	20.52	0.72 (0.61–0.84)	16.00	0.56 (0.46–0.68)
			1989–1998	22.92	14.87	0.65 (0.54–0.78)	12.27	0.54 (0.43–0.67)
		Female	1979–1988	7.55	4.80	0.64 (0.46–0.87)	4.44	0.59 (0.41–0.84)
			1989–1998	4.76	4.53	0.95 (0.67–1.36)	3.99	0.84 (0.55–1.27)
	65– ≥ 85	Male	1979–1988	543.74	468.55	0.86 (0.81–0.91)	455.07	0.84 (0.79–0.89)
			1989–1998	321.46	313.94	0.98 (0.91–1.05)	279.38	0.87 (0.80–0.94)
		Female	1979–1988	335.99	286.01	0.85 (0.80–0.91)	291.62	0.87 (0.81–0.93)
			1989–1998	193.20	185.38	0.96 (0.89–1.03)	161.30	0.83 (0.77–0.91)
Diabetes mellitus (250.0–250.9)								
	45– ≥ 85	Male	1979–1988	32.60	NA	NA	36.28	1.11 (0.98–1.26)
			1989–1998	44.79	NA	NA	55.73	1.24 (1.13–1.37)
		Female	1979–1988	28.75	NA	NA	30.19	1.05 (0.93–1.18)
			1989–1998	35.83	NA	NA	42.03	1.17 (1.06–1.30)
	45–64	Male	1979–1988	13.15	NA	NA	13.35	1.01 (0.76–1.35)
			1989–1998	15.00	NA	NA	18.77	1.25 (0.97–1.62)
		Female	1979–1988	8.85	NA	NA	8.70	0.98 (0.70–1.39)
			1989–1998	9.87	NA	NA	11.95	1.21 (0.88–1.66)
	65– ≥ 85	Male	1979–1988	73.13	NA	NA	84.07	1.15 (1.01–1.31)
			1989–1998	106.86	NA	NA	132.72	1.24 (1.12–1.38)
		Female	1979–1988	70.22	NA	NA	74.98	1.07 (0.95–1.21)
			1989–1998	89.94	NA	NA	104.72	1.16 (1.05–1.29)

NA, not applicable.
